# Real‐life benefits of intrajejunal levodopa infusion therapy in four patients with the parkinsonian variant of progressive supranuclear palsy: A 1‐year follow‐up data report

**DOI:** 10.1002/brb3.2547

**Published:** 2022-08-09

**Authors:** Vinod Metta, K. Ray Chaudhuri, Valentina Leta, Kandadai Rukmini Mridula, Chaitanya Koduri, Sai Deepak, Rakesh Kalpala, Nageswar Reddy, Guy Chung‐Faye, Rupam Borgohain

**Affiliations:** ^1^ Department of Basic and Clinical Neurosciences Institute of Psychiatry Psychology & Neuroscience King's College London London UK; ^2^ Parkinson's Foundation Centre of Excellence King's College Hospital London UK; ^3^ Nizams Institute of Medical Sciences Hyderabad India; ^4^ Asian Institute of Gastroenterology Hyderabad India; ^5^ Department of Gastroenterology King's College Hospital London UK

**Keywords:** intrajejunal levodopa‐carbidopa infusion, motor and nonmotor symptoms, Parkinsonian variant of progressive supranuclear palsy, quality of life

## Abstract

**Background:**

Progressive supranuclear palsy (PSP) is a progressive neurodegenerative condition presenting with different clinical endophenotypes. The parkinsonian variant of PSP (PSP‐P) is characterised by early but fading responsiveness to high‐dose levodopa therapy; however, high‐dose oral therapy is often associated with intolerance due to dopaminergic side effects and so doses may have to be capped despite clinical benefits. Evidence from animal models and real‐life registries suggest far higher doses of levodopa can be tolerated if given in a continuous drug delivery (CDD) manner. We investigated tolerance and possible clinical benefits in patients with PSP‐P still responsive to levodopa after initiating CDD in the form of intrajejunal levodopa infusion (IJLI) therapy as part of a compassionate usage program (CU).

**Methods:**

This is an observational clinical data report from the IJLI implementation program undertaken in regional tertiary referral Parkinson's centres in India and at King's College Hospital London, Dubai as part of a CU. Four patients with PSP‐P receiving IJLI as a part of a CU underwent evaluations of liver and renal function, motor and nonmotor function, quality of life, sleep dysfunction, fatigue, anxiety and depression, and cognitive impairment at baseline and 6 and 12 months post‐IJLI initiation.

**Results:**

In total, three out of four patients successfully completed 12 months of treatment (6 months in one patient). All four patients showed good tolerability to IJLI even at higher doses (1400 and 1960 mg at 6 and 12 months, respectively) when compared to oral levodopa (812.5 ± 103 levodopa equivalent daily dose [LEDD]) and presented with overall persistent improvements in motor and nonmotor scores and quality‐of‐life scores at 6 and 12 months post‐IJLI. All patients showed improvement in estimated glomerular filtration rate (43.50 ml/min/1.73 m^2^ to 67.5 ml/min/1.73 m^2^ and 79.5 ml/min/1.73 m^2^ at 6 and 12 months, respectively).

**Conclusions:**

IJLI led to persistent beneficial effects on motor and some nonmotor aspects in patients with PSP‐P at up to 12 months after treatment with associated improvement in overall renal function.

## Graphical abstract

Intrajeujunal levodopa infusion for patients with Progressive supranuclear palsy‐Parkinson's variant

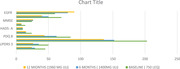

## 1 INTRODUCTION

Progressive supranuclear palsy (PSP, or Steele–Richardson–Olszewski syndrome) is a progressive neurodegenerative form of tauopathy first described by Steele, Richardson, and Olszewski in 1964. PSP affects movement, swallowing, speech, gait, eye movements, mood, behavior, and cognition and results in problems such as supranuclear gaze palsy, progressive axial rigidity, pseudobulbar palsy, and mild dementia, all of which are linked to the severity of abnormal tau accumulation and neuronal loss in various brain regions (Steele, 1972; Steele et al., 1964). PSP characteristically occurs between 60 and 65 years of age. In 2017, the International Parkinson and Movement Disorder Society published the Movement Disorders Society‐Progressive Supranuclear Palsy (MDS‐PSP) criteria covering a spectrum of phenotypes and a number of clinical subtypes, including classic progressive supranuclear palsy‐Richardson syndrome (PSP‐RS), progressive supranuclear palsy‐parkinsonism (PSP‐P), progressive supranuclear palsy‐pure akinesia with gait freezing (PSP‐PAGF), progressive supranuclear palsy‐corticobasal syndrome (PSP‐CBS), progressive supranuclear palsy‐behavioural variant of frontotemporal dementia (PSP‐bvFTD), and progressive supranuclear palsy‐progressive nonfluent aphasia (PSP‐PNFA) (Höglinger et al., 2017).

To date, studies of potentially disease‐modifying therapies have failed to demonstrate obvious efficacy in individuals suspected to have PSP. For motor (parkinsonian) symptoms, levodopa with a DOPA decarboxylase inhibitor (e.g., carbidopa or benserazide) is generally prescribed; this combination typically shows modest to no success in most PSP phenotypes, but it may be potentially beneficial in the PSP‐P subtype, particularly at higher doses (Rowe et al., 2021). However, because of the limited therapeutic options and associated side effects, levodopa is generally orally administered at doses of up to 1000 mg daily and possible beneficial oral higher doses are often not tolerated (Rowe et al., 2021). As such, therapy for PSP, and specifically PSP‐P, remain limited to largely palliative care given the inexorable progression of this condition.

Initial levodopa responsiveness in some patients with PSP‐P, however, is cause of some optimism and in some cases can be considerable. Intrajejunal levodopa infusion (IJLI) allows levodopa to be administered and tolerated at a much higher dose as global registry data from patients with PD would indicate (Antonini et al., 2017; Standaert et al., 2020). Other therapeutic options that have been attempted include a report by Scelzo et al. (2017) who performed unilateral and bilateral pedunculopontine nucleus (PPN) deep brain stimulation (DBS) in patients with suspected PSP; in eight individuals with PSP‐RS, the treatment offered no benefits in gait and postural stability, and DBS is currently not recommended for progressive supranuclear palsy outside of research settings (Scelzo et al., 2017). No other device‐aided nonoral infusion therapies (DAT), such as apomorphine or IJLI infusion therapies, (Metta et al., 2021) indicated in patients with advanced Parkinson's disease (APD), have been used in patients with PSP‐P. IJLI provides continuous dopamine delivery through the jejunum and there is reasonable evidence to suggest that levodopa administered by the intrajejunal route can be given by far higher doses (Jenner, 2015). Here, we report the findings for four patients who were diagnosed with PSP‐P and received IJLI on a compassionate usage program with clinical follow‐up assessments at baseline and 6 and 12 months.

## 2 METHODS

This is an observational clinical data report from the IJLI Implementation Program undertaken in regional tertiary referral Parkinson's centres in India and at King's College Hospital London, Dubai as part of a compassionate usage program (CU). A CU is defined by the World Health Organization as a program that is intended to provide potentially life‐saving treatments to patients suffering from a disease for which no satisfactory authorised therapy exists and/or who cannot enter a clinical trial, or use of drugs that are approved in one country but not available globally; for many patients, these programs represent their last hope. In India, CUs are regulated by the Central Drugs Standard Control Organization (CDSCO) according to Health Regulatory Act rules 33A and 34A of the 1940 Drugs and Cosmetic Act, 1940 and Rules, 1945 that allows import of small quantities of new drugs by a government hospital or autonomous medical institution for the treatment of patients suffering from life‐threatening diseases or diseases causing serious permanent disability, or such disease requiring therapies for unmet medical needs (The Drugs and Cosmetics Act, 1940 (23 of 1940) and the Drugs and Cosmetics Rules 1945, 2016).

Four patients with PSP‐P receiving IJLI as a part of a CU underwent evaluations of liver and renal function, motor (Unified Parkinson's Disease Rating Scale III [UPDRS III]) and nonmotor (Nonmotor Symptoms Scale [NMSS]) function, quality of life (Parkinson's Disease Questionnaire 8 [PDQ‐8]), sleep dysfunction (Parkinson's Disease Sleep Scale [PDSS]), fatigue (Parkinson's Disease Fatigue Scale 16 [PFS 16]), anxiety and depression (Hospital Anxiety and Depression Scale [HADS]), and cognitive impairment (Mini Mental State Examination [MMSE]) at baseline and 6 and 12 months post‐IJLI initiation. Descriptive statistics were used for continuous variables and frequency for categorial data.

## 3 RESULTS

### 3.1 Patient 1

A 71‐year‐old Indian man had been diagnosed with parkinsonism 6 years previously when he presented with unexplained slowness and stiffness, which affected his writing and walking. He showed an initial beneficial response to dopaminergic treatment, but 3 years later, his speech worsened, and he experienced some falls. His oral dopamine dosage was increased, but his overall clinical condition did not improve for a year, after which he began to experience mild swallowing problems. The dysphagia work‐up showed normal findings, and his oral dopaminergic treatment was further optimized. However, he began to show reduced tolerance to the increasing levodopa dosage and showed hallucinations, progressive decline in overall clinical condition, worsening gait, falls, inability to get up from a chair, difficulties chewing and swallowing, and a range of nonmotor symptoms (including constipation, urinary problems such as incontinence, sleep‐related problems). His other medical problems included type 2 diabetes that was well‐controlled with metformin monotherapy (1 g), essential hypertension that was treated with 5 mg captopril. There was no other significant past medical history, no family history of dementia and no history of allergies.

The patient was diagnosed with PSP‐P. Since he was a retired medical practitioner, his family included other physicians who were aware about his condition, and due to the lack of obvious definitive treatment options available, IJLI was considered under a CU based on his initial response to oral levodopa.

### 3.2 Patient 2

A 61‐year‐old Indian man presented with symptoms suggestive of parkinsonism and was diagnosed with Parkinson's Plus disease (PSP‐P) 5 years ago. The patient had a history of hypertension that was well‐controlled with 5 mg amlodipine and reported no other significant past medical history. He developed adverse reactions against an initial dopamine agonist regimen (dopamine dysregulation syndrome [DDS] on treatment with pramipexole, and severe somnolence issues on treatment with ropinirole). Subsequently, he showed good initial response to levodopa treatment; however, the treating clinicians reported no optimal response to an increased dosage in the oral dopaminergic regime. In addition to cardiovascular, urinary, and gastrointestinal dysfunction, the patient showed severe sleep‐related issues and other problems, including symptoms suggestive of restless leg syndrome (RLS).

Considering a diagnosis of PSP and the lack of obvious definitive treatment options, based on his initial response to oral levodopa and the patient's and his family's request based on the initial beneficial response to oral levodopa, IJLI was considered under a CU.

### 3.3 Patient 3

A 70‐year‐old Indian man with history of long‐term type 2 diabetes and essential hypertension for the last 20 years presented with unilateral onset of left‐sided upper limb tremors, stiffness, and slowness. The initial diagnosis was idiopathic Parkinson's disease since the DAT results were positive, and the patient was started on levodopa, which showed a beneficial response initially. However, he showed progressive worsening of gait, speech, and swallowing functions despite optimization of his levodopa regimen and was subsequently unable to tolerate the increasing oral pulsed and frequent dosing. He showed hallucinations, dyskinesia, and falls, and was subsequently diagnosed with PSP‐P. After a subsequent discussion of all treatment options with the patient, IJLI was considered under a CU.

### 3.4 Patient 4

A 74‐year‐old Indian man with a 9‐year history of progressive worsening of balance, falls, and speech and swallowing problems, presented with right‐sided stiffness and mild tremors. He showed a beneficial response to a levodopa regimen, but subsequently showed an inability to tolerate increasing levodopa doses and developed adverse reactions to dopamine agonists. His medical history included RLS treated with 1 mg clonazepam daily and type 2 diabetes treated with 1 g metformin daily. He had no other significant family history. We diagnosed PSP‐P and IJLI was considered on a CU after discussion all treatment options.

The mean age of these patients was 69 ± 4.9 years, the mean disease duration was 6 ± 0.86 years, and the mean total oral levodopa‐equivalent daily dose was 812.5 ± 103 mg. All 4 patients showed an initial good response to dopaminergic regimens but were unable to tolerate escalation of levodopa because of adverse effects and gastrointestinal issues, frequent falls, worsening mobility (baseline mean scores: UPDRS 2, 44; UPDRS 3, 49; NMSS, 213.75; PDQ‐8, 84.2; HADS (D), 23.5; HADS (A), 14; PFS‐16, 14.5; PDSS, 76.5; MMSE, 24; eGFR, 43.5).

**FIGURE 1:**
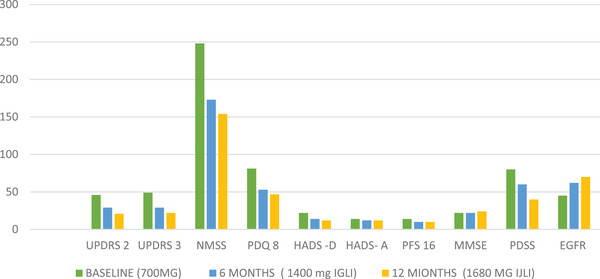
Scale based assessments, results for Patient 1 performed at an optimally on condition. IJLI, intrajejunal levodopa infusion therapy; eGFR, estimated glomerular filtration rate; HADS, Hospital Anxiety and Depression Scale; MMSE, Mini Mental State Examination; NMSS, Nonmotor Symptoms Scale; PDQ, Parkinson's Disease Questionnaire; PDSS, Parkinson's Disease Sleep Scale; PFS, Parkinson's Fatigue Scale; UPDRS, Unified Parkinson's Disease Rating Scale

**FIGURE 2:**
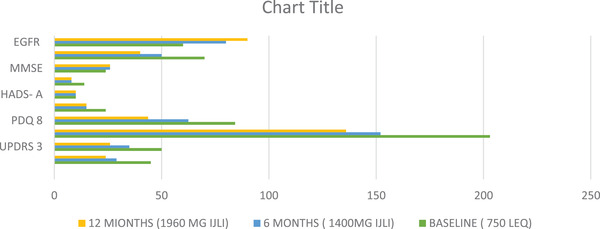
Scale based assessments,results for Patient 2 performed at an optimally on condition. eGFR, estimated glomerular filtration rate; HADS, Hospital Anxiety and Depression Scale; IJLI, intrajejunal levodopa infusion therapy; LED, levodopa equivalent dose; MMSE, Mini Mental State Examination; PDQ, Parkinson's Disease Questionnaire; UPDRS, Unified Parkinson's Disease Rating Scale

**FIGURE 3:**
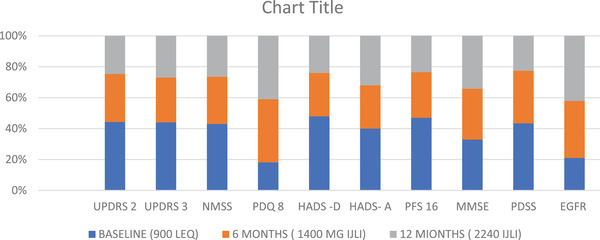
Scale based assessments for Patient 3 performed at an optimally on condition. eGFR, estimated glomerular filtration rate; HADS, Hospital Anxiety and Depression Scale; IJLI, intrajejunal levodopa infusion therapy; MMSE, Mini Mental State Examination; NMSS, Nonmotor Symptoms Scale; PDQ, Parkinson's Disease Questionnaire; PDSS, Parkinson's Disease Sleep Scale; PFS, Parkinson's Fatigue Scale; UPDRS, Unified Parkinson's Disease Rating Scale

**FIGURE 4:**
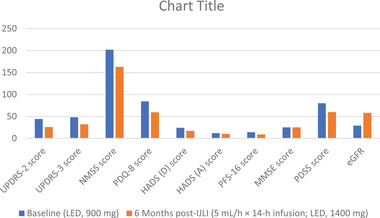
Scale based assessments for Patient 4 performed at an optimally on condition. eGFR, estimated glomerular filtration rate; HADS, Hospital Anxiety and Depression Scale; IJLI, intrajejunal levodopa infusion therapy; LED, levodopa equivalent dose; MMSE, Mini Mental State Examination; NMSS, Nonmotor Symptoms Scale; PDQ, Parkinson's Disease Questionnaire; PDSS, Parkinson's Disease Sleep Scale; PFS, Parkinson's Fatigue Scale; UPDRS, Unified Parkinson's Disease Rating Scale.

For all four patients, IJLI via a percutaneous endoscopic intragastric jejunostomy (PEG‐J) was initiated on compassionate grounds, and as a good clinical practice, initial titration was started with a maintenance dose of (5 ml/h (100 mg of LEDD)) (Tomlinson et al., 2010) with a 14‐h infusion with overnight levodopa and carbidopa (200/50) control release was started and discharged was maintained on this regimen for 6 months maintained dose of (LEDD 1400 mg) was tolerated with no immediate complications. At 12 months, the ILJI maintenance dosage was escalated to 7 ml/h (1960 mg LEDD). All four patients, even at 12 months, were able to tolerate increased levodopa given intrajejunally (1960 ± 203 mg LEDD) when compared to an oral tolerable dose of 812.5 ± 103 mg. There were no immediate complications associated with escalation apart from a dislodged tube that was fixed immediately; all patients tolerated levodopa infusion and showed better outcomes, with an overall improvement of 34%–41% in UPDRS motor scores and NMSS scores, 37%–40% improvement in PDQ‐8 and HADS scores, and 40%–45% improvement in fatigue and sleep scores. Renal function also improved, with the eGFR increasing from 43.5% to 67.5% at 6 months and 79.5% at 12 months, indicating that patients were adequately hydrated and fed through the dual‐port PEG‐J. The memory scores also remained stable (Tables 1, 2, 3, 4 and Figures 1, 2, 3, 4).

**TABLE 1:** Scale based assessments, results for Patient 1 performed at an optimally on condition

	Baseline (LED, 700 mg)	Six months post‐IJLI (5 ml/h × 14 h infusion; LED, 1400 mg)	Twelve months post‐IJLI (6 ml/h; LED, 1680 mg)
UPDRS part 2 score	46	29	21
UPDRS part 3 score	49	29	29
NMSS score	248	173	154
PDQ‐8 score	81.25	53.12	46.8
HADS (D) score	22	14	12
HADS (A) score	14	12	12
PFS‐16 score	14	10	10
MMSE score	22	22	24
PDSS score	80	60	50
eGFR	45	62	70

*Abbrevations*: eGFR, estimated glomerular filtration rate; HADS, Hospital Anxiety and Depression Scale; IJLI, intrajejunal levodopa infusion therapy; LED, levodopa equivalent dose; MMSE, Mini Mental State Examination; NMSS, Nonmotor Symptoms Scale; PDQ, Parkinson's Disease Questionnaire; PDSS, Parkinson's Disease Sleep Scale; PFS, Parkinson's Fatigue Scale; UPDRS, Unified Parkinson's Disease Rating Scale.

**TABLE 2:** Scale based assessments, results or Patient 2 performed at an optimally on condition

	Baseline (LED, 750 mg)	Six months post‐IJLI (5 ml/h × 14 h infusion; LED, 1400 mg)	Twelve months post‐IJLI (7 ml/h × 14 h infusion; LED, 1960 mg)
UPDRS‐2 score	45	29	21
UPDRS‐3 score	50	35	22
NMSS score	203	152	136
PDQ‐8 score	84.3	62.5	43.75
HADS (D) score	24	15	15
HADS (A) score	10	10	10
PFS‐16 score	14	8	8
MMSE score	24	26	26
PDSS score	70	50	40
eGFR	60	80	90

*Abbrevations*: eGFR, estimated glomerular filtration rate; HADS, Hospital Anxiety and Depression Scale; IJLI, intrajejunal levodopa infusion therapy; LED, levodopa equivalent dose; MMSE, Mini Mental State Examination; NMSS, Nonmotor Symptoms Scale; PDQ, Parkinson's Disease Questionnaire; PDSS, Parkinson's Disease Sleep Scale; PFS, Parkinson's Fatigue Scale; UPDRS, Unified Parkinson's Disease Rating Scale.

**TABLE 3:** Scale based assessments, results for Patient 3 performed at an optimally on condition

	Baseline (LED, 900 mg)	Six months post‐IJLI (5 ml/h × 14 h infusion; LED, 1400 mg)	Twelve months post‐IJLI (8 ml/h × 14 h infusion; LED, 2240 mg)
UPDRS‐2 score	43	30	24
UPDRS‐3 score	49	32	30
NMSS score	202	143	124
PDQ‐8 score	84.3	56.25	56.25
HADS (D) score	24	14	12
HADS (A) score	20	14	16
PFS‐16 score	16	10	8
MMSE score	25	25	26
PDSS score	77	60	50
eGFR	40	70	80

*Abbrevations*: eGFR, estimated glomerular filtration rate; HADS, Hospital Anxiety and Depression Scale; IJLI, intrajejunal levodopa infusion therapy; LED, levodopa equivalent dose; MMSE, Mini Mental State Examination; NMSS, Nonmotor Symptoms Scale; PDQ, Parkinson's Disease Questionnaire; PDSS, Parkinson's Disease Sleep Scale; PFS, Parkinson's Fatigue Scale; UPDRS, Unified Parkinson's Disease Rating Scale.

**TABLE 4:** Scale based assessments, results for Patient 4 performed at an optimally on condition

	Baseline (LED, 900 mg)	Six months post‐IJLI (5 ml/h × 14 h infusion; LED, 1400 mg)
UPDRS‐2 score	44	26
UPDRS‐3 score	48	32
NMSS score	202	163
PDQ‐8 score	84.3	59.3
HADS (D) score	24	17
HADS (A) score	12	10
PFS‐16 score	14	9
MMSE score	25	25
PDSS score	80	60
eGFR	29	58

*Abbrevations*: eGFR, estimated glomerular filtration rate; HADS, Hospital Anxiety and Depression Scale; IJLI, intrajejunal levodopa infusion therapy; LED, levodopa equivalent dose; MMSE, Mini Mental State Examination; NMSS, Nonmotor Symptoms Scale; PDQ, Parkinson's Disease Questionnaire; PDSS, Parkinson's Disease Sleep Scale; PFS, Parkinson's Fatigue Scale; UPDRS, Unified Parkinson's Disease Rating Scale.

**FIGURE 5:**
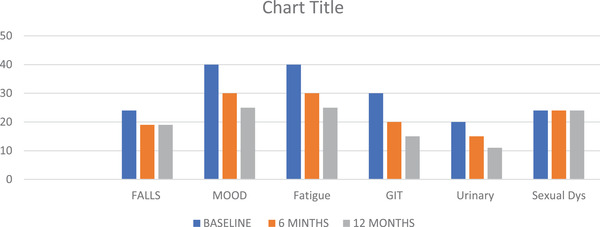
Effects of IJLI on individual NMSS domains. GIT, gastrointestinal symptoms; IJLI, intrajejunal levodopa infusion therapy; NMSS, Nonmotor Symptoms Scale

**FIGURE 6:**
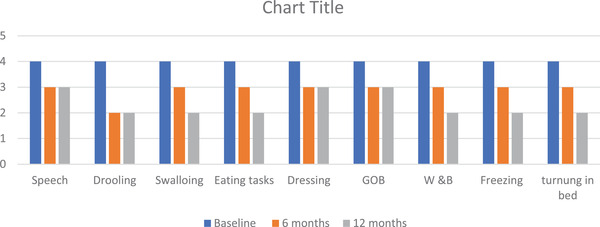
Effects of IJLI on individual UPDRS‐2 domains. IJLI, intrajejunal levodopa infusion therapy; UPDRS, Unified Parkinson's Disease Rating Scale (GOB = Getting out of Bed) W&B = Walking & Balance)

**FIGURE 7:**
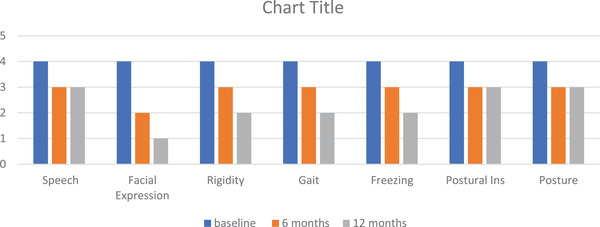
Effects of IJLI on individual UPDRS‐3 domains. IJLI, intrajejunal levodopa infusion therapy; UPDRS, Unified Parkinson's Disease Rating Scale

The nonmotor domain data shown in Figure 5 indicated an overall improvement in gastrointestinal symptoms, mood, fatigue, and fall domains, but the patients showed no improvement in the sexual function and motor domains (Figures 6 and 7). The patients showed a persistent improvement in drooling and swallowing at 6 and 12 months (Figure 6), and although a 25%–30% improvement was observed in the speech and postural instability domains initially, the improvements plateaued from 6 to 12 months. The overall quality of life as measured by the PDQ‐8 continued to improve from baseline to 6 months (37%‐40%), and at 12 months, the overall scores had increased further by 5%–10% to yield an overall improvement of 40%–45%.

## 4 DISCUSSION

We describe the persistent beneficial effects of IJLI in patients with PSP‐P, who continued to report improvements in motor and some nonmotor aspects and overall quality of life even 6 and 12 months after IJLI and showed an improvement in overall renal function post‐IJLI. While our study has limitations, like the small sample size and short‐term follow‐up, our results highlight better tolerability and responsiveness to intrajejunal levodopa compared to the conventional pulsatile regimen.

## FUNDING

None.

## CONFLICT OF INTEREST

The authors declare no conflicts of interest.

## AUTHOR CONTRIBUTIONS

All authors contributed to drafting and editing the manuscript. The corresponding author oversaw the entire implementation process.

### PEER REVIEW

The peer review history for this article is available at https://publons.com/publon/10.1002/brb3.2547

## Data Availability

Raw data were generated at Kings College hospital London, Dubai . Derived data supporting the findings of this study are available from the corresponding author VM on request.
